# Engineering natural *Saccharomyces cerevisiae* isolates for enhanced one-step cellulosic ethanol production

**DOI:** 10.1007/s00253-026-13830-0

**Published:** 2026-05-08

**Authors:** Letitia Sabina Minnaar, Kentaro Inokuma, Tomohisa Hasunuma, Riaan den Haan

**Affiliations:** 1 Institute for Microbial Biotechnology and Metagenomics, Department of Biotechnology, University of the Western Cape, Bellville, 7530 South Africa; 2 Graduate School of Science, Technology and Innovation, Kobe University, 1-1 Rokkodai-Cho, Nada-Ku, Kobe, 657-8501 Japan; 3 Engineering Biology Research Center, Kobe University, 1-1 Rokkodai-Cho, Nada-Ku, Kobe, 657-8501 Japan; 4 Department of Microbiology, Stellenbosch University, Stellenbosch, 7600 South Africa

**Keywords:** Cell-tethered expression, *Saccharomyces cerevisiae*, Secretion, Cellulases, Natural isolates, Consolidated bioprocessing

## Abstract

**Abstract:**

Engineering yeast strains for use as chassis organisms in second-generation (2G) bioethanol is a promising strategy to improve process economics. Natural isolates of *Saccharomyces cerevisiae* offer strain backgrounds with greater genetic diversity and enhanced robustness, with the potential for improved heterologous protein production capabilities. In this study, heterologous cellulase production using different expression strategies was evaluated in various process-relevant conditions. Enhanced cellulolytic activity was clearly demonstrated in a cell-tethered enzyme system, compared to a free enzyme system, across identical strain backgrounds. Superior secretory capacity was obtained for YI59_V2 for all individual enzymes across all process-relevant conditions tested. In addition, this strain exhibited improved hydrolysis efficiency and ethanol production from crystalline cellulose, achieving ~10 g/L after 96 h (~88% of the maximum theoretical yield) without the need for exogenous cellulase supplementation. Interestingly, enhanced strain robustness against process-relevant, secretion, and cell wall stresses was also observed in transformants with cell-tethered cellulase systems compared to those with free enzyme systems. This study highlights that the expression design strategy for cellulase-encoding genes in this natural isolate was pivotal for increasing protein titres and for influencing strain robustness. Strains exhibiting elevated cellulase activity and increased robustness represent a key step toward the industrial deployment of consolidated bioprocessing (CBP).

**Key points:**

• *Cell-tethered expression greatly boosted cellulase activity and cellulose breakdown.*

• *YI59_V2 yielded ~ 10 g/L ethanol from crystalline cellulose without added enzymes.*

• *Tethered enzymes reshaped cell walls and altered stress tolerance.*

**Supplementary Information:**

The online version contains supplementary material available at 10.1007/s00253-026-13830-0.

## Introduction

Producing second-generation (2G) bioethanol on an industrial scale has drawn much attention over the past few decades (Audibert et al. 2025). Not only would 2G bioethanol contribute significantly to ensuring sustainability and energy security, but waste disposal management would also be enhanced (Olguin-Maciel et al. 2020). However, the current routes used for 2G bioethanol production are based on the use of costly pretreatment methods to assist in degrading the recalcitrant structure of the lignocellulosic biomass feedstocks (Abo et al. 2019; Valuenzuela-Ortega and French 2019; Zoghlami and Paës, 2019). While effective, these methods introduce inhibitory components and/or conditions that often hold detrimental effects for the normal growth and metabolism of fermentative organisms (Cheah et al. 2020; Sharma et al. 2022). In addition, industrial applications require the use of exogenous cellulase cocktails for feedstock hydrolysis, which costs up to 40% of the entire production process (Branco et al. 2019; Dadwal et al. 2020). As such, continuous research efforts are exploring engineering avenues to confer cellulolytic capabilities to natural ethanologenic organisms that lack the inherent ability to produce these cellulases (Borthakur et al. 2024).

An organism that has several advantages for 2G bioethanol production is the budding yeast *Saccharomyces cerevisiae* (Brandt et al. 2021). Not only does *S. cerevisiae* have generally regarded as safe (GRAS) status, but it also exhibits robustness to a multitude of stresses, high ethanol-producing capabilities, fast growth in cheap media, and amenability to genetic engineering (Fernandes et al. 2022; Zhao et al. 2024). As an added advantage, the greater phenotypic variation and diversity offered by natural isolates of *S. cerevisiae* has paved the way for obtaining even better hosts for 2G bioethanol production processes compared to domesticated industrial strains (Corbu and Csutak 2023; Villarreal et al. 2025). Since natural isolates inhabit environments that greatly overlap with industrial bioethanol conditions, strains have evolved to attain greater genetic complexity and phenotypic variation that is beneficial for their survival in harsh conditions (De Witt et al. 2019). Since high heterozygosity is often displayed by these strains, a superior foundation therefore exists for precision genetic engineering, which would greatly benefit the enhancement of heterologous cellulolytic activity in these hosts.

The release of fermentable glucose from the glucose-based polysaccharide cellulose requires the synergistic action of at least an endoglucanase (EG), a cellobiohydrolase (CBH), and a β-glucosidase (BGL). While significant progress has been made pertaining to expression of such a core set of cellulases in *S. cerevisiae*, using different strategies including secretion (Liu et al. 2024; Minnaar and Den Haan 2023), cell-tethering (Chetty et al. 2022; Inokuma et al. 2021), and/or mini-cellulosome construction (Ma et al. 2024), the enzyme titres produced and the substrate conversion efficiencies attained remained far below the required levels necessary for industrial applications (Dadwal et al. 2020).

Recently, research efforts into improving cellulase production identified a novel transcriptional regulator *MDG1* capable of not just enhancing heterologous cellulase production, but also shifting the optimal catalytic temperature of BGL1 (Wan et al. 2025). In another study, overexpression of the *LAS21* gene involved in glycosylphosphatidylinositol (GPI) biosynthesis was shown to enhance BGL1 activity by more than 48% when cell-tethered in *S. cerevisiae* (Arnthong et al. 2022). Similarly, with promoter engineering approaches, Deng and co-workers showed that fine-tuning of *GAL* promoters could enhance promoter strengths roughly twofold (Deng et al. 2021). Combinations of promoters and proximal introns were also shown to enhance amylase activity in starch-hydrolyzing *S. cerevisiae* strains (Schwerdtfeger et al. 2023). These studies show that considerable potential exists to enhance cellulase production, using genetic engineering approaches, particularly with promoters and terminators more suitable to the fermentation environment.

In this study, natural isolates of *S. cerevisiae* were engineered to express a core set of cellulase encoding genes under the control of stress-induced promoters and terminators, with the enzymes subsequently tethered to the cell wall. In addition, secretion-based isolates of *S. cerevisiae* constructed in a prior study (Minnaar and Den Haan 2023) were included for comparison. Transformants that were superior secretors of cell-tethered *Trichoderma reesei* endoglucanase II, *Talaromyces emersonii* cellobiohydrolase I, *Aspergillus aculeatus* $\beta$-glucosidase I, and *Chrysosporium lucknowense* cellobiohydrolase II were identified using individual colorimetric and/or fluorometric activity assay screenings, after successive rounds of transformations. Tolerance profiles and growth kinetics were further evaluated to compare phenotypic differences between wild-type, secretion-, and cell-tethered-based strain isolates. Finally, superior strains were evaluated for their ability to directly convert microcrystalline cellulose (Avicel) to ethanol, without the addition of exogenous cellulase cocktails. Our results demonstrated that the expression strategy used for cellulase gene expression played a vital role in the resulting substrate hydrolysis and ethanol titres obtained. Furthermore, these isolates showed considerable promise as chassis organisms for advancing industrial bioethanol production processes utilizing lignocellulosic biomass.

## Methods

### Microbial strain cultivations

All chemicals, reagents, and media components used were of laboratory grade and purchased from Sigma/Merck (St. Louis, MO, USA), unless otherwise stated. Microbial yeast strains (Table 1) were streaked from 15% (v/v) glycerol stocks stored at −80 °C onto YPD agar (1% yeast extract, 2% peptone, 2% D-glucose, and 2% bacteriological agar) medium supplemented with 100 µg.mL^−1^ CloNAT (Jena Bioscience, Jena, Germany) and/or 200 µg.mL^−1^ Geneticin G418 (Invitrogen, Waltham, MA, USA) as required, followed by incubation at 30 °C for 48–72 h to obtain single colonies. Liquid YPD broth, with or without antibiotic selection, was then inoculated with single colonies of yeast cultures, followed by overnight incubation at 30 °C on an orbital shaker spun at 180 rpm.

**Table 1:** Microbial strains used in this study

Microbial strain	Abbreviation	Description	Reference
Parental strains			
*S. cerevisiae* YI13	YI13_WT	Natural isolate	Davison et al. (2016)
*S. cerevisiae* YI59	YI59_WT	Natural isolate	Davison et al. (2016)
*S. cerevisiae* MH1000	MH1000_WT	Diploid industrial strain with no auxotrophy	Davison et al. (2016)
*S. cerevisiae* Ethanol Red® (ER)	ER_WT	Diploid industrial strain with no auxotrophy	Davison et al. (2016)
Constructed strains			
*S. cerevisiae* YI13 + pCas9-NAT + (pRS42-G-ChX + *ENO*1_P_-*T.r.eg2*-*ENO*1_T_) + (pRS42-G-$\Delta$ + *ENO*1_P_-*T.e.cbh1*-*ENO*1_T_) + (pRS42-G-ChXI + *SED*1_P_-SS-*A.a.bgl1*-AD-*DIT*1_T_) + (pRS42-G-$\Delta$ + *ENO*1_P_-*C.l.cbh2*-*ENO*1_T_)	YI13_BECC (YI13_V1)*	YI13_WT transformed with CRISPR plasmids, pCas9-NAT and sgRNA plasmids (pRS42-G-ChX, pRS42-G-ChXI, and pRS42-G-$\Delta$, respectively) and the homology repair templates *T.r.eg2*, *A.a.bgl1*, *T.e.cbh1*, and *C.l.cbh2*, respectively, in successive rounds of transformation	Minnaar and den Haan (2023)
*S. cerevisiae* YI59 + pCas9-NAT + (pRS42-G-ChX + *ENO*1_P_-*T.r.eg2*-*ENO*1_T_) + (pRS42-G-$\Delta$ + *ENO*1_P_-*T.e.cbh1*-*ENO*1_T_) + (pRS42-G-ChXI + *SED*1_P_-SS-*A.a.bgl1*-AD-*DIT*1_T_) + (pRS42-G-$\Delta$ + *ENO*1_P_-*C.l.cbh2*-*ENO*1_T_)	YI59_BECC (YI59_V1)*	YI59_WT transformed with CRISPR plasmids, pCas9-NAT and sgRNA plasmids (pRS42-G-ChX, pRS42-G-ChXI, and pRS42-G-$\Delta$, respectively) and the homology repair templates *T.r.eg2*, *A.a.bgl1*, *T.e.cbh1*, and *C.l.cbh2*, respectively, in successive rounds of transformation	Minnaar and den Haan (2023)
*S. cerevisiae* MH1000 + pCas9-NAT + (pRS42-G-ChX + *ENO*1_P_-*T.r.eg2*-*ENO*1_T_) + (pRS42-G-$\Delta$ + *ENO*1_P_-*T.e.cbh1*-*ENO*1_T_) + (pRS42-G-ChXI + *SED*1_P_-SS-*A.a.bgl1*-AD-*DIT*1_T_) + (pRS42-G-$\Delta$ + *ENO*1_P_-*C.l.cbh2*-*ENO*1_T_)	MH1000_BECC (MH1000_V1)*	MH1000_WT transformed with CRISPR plasmids, pCas9-NAT and sgRNA plasmids (pRS42-G-ChX, pRS42-G-ChXI, and pRS42-G-$\Delta$, respectively) and the homology repair templates *T.r.eg2*, *A.a.bgl1*, *T.e.cbh1*, and *C.l.cbh2*, respectively, in successive rounds of transformation	Minnaar and den Haan (2023)
*S. cerevisiae* YI13 + pCas9-NAT + (pRS42-G-ChX + *SED*1_P_-SS-*T.r.eg2*-AD-*DIT*1_T_) + (pRS42-G-$\Delta$ + *SED*1_P_-SS-*T.e.cbh1*-AD-*DIT*1_T_) + (pRS42-G-ChXI + *SED*1_P_-SS-*A.a.bgl1*-AD-*DIT*1_T_) + (pRS42-G-CAN1 + *SED*1_P_-SS-*C.l.cbh2*-AD-*DIT*1_T_)	YI13_V2	YI13_WT transformed with CRISPR plasmids, pCas9-NAT and sgRNA plasmids (pRS42-G-ChX, pRS42-G-ChXI, and pRS42-G-$\Delta$, pRS42-G-CAN1, respectively) and the homology repair templates *T.r.eg2*, *A.a.bgl1*, *T.e.cbh1*, and *C.l.cbh2*, respectively, in successive rounds of transformation	This study
*S. cerevisiae* YI59 + pCas9-NAT + (pRS42-G-ChX + *SED*1_P_-SS-*T.r.eg2*-AD-*DIT*1_T_) + (pRS42-G-$\Delta$ + *SED*1_P_-SS-*T.e.cbh1*-AD-*DIT*1_T_) + (pRS42-G-ChXI + *SED*1_P_-SS-*A.a.bgl1*-AD-*DIT*1_T_) + (pRS42-G-ChXII + *SED*1_P_-SS-*C.l.cbh2*-AD-*DIT*1_T_)	YI59_V2	YI59_WT transformed with CRISPR plasmids, pCas9-NAT and sgRNA plasmids (pRS42-G-ChX, pRS42-G-ChXI, and pRS42-G-$\Delta$, and pRS42-G-ChXII, respectively) and the homology repair templates *T.r.eg2*, *A.a.bgl1*, *T.e.cbh1*, and *C.l.cbh2*, respectively, in successive rounds of transformation	This study
*S. cerevisiae* ER + pCas9-NAT + (pRS42-G-ChX + *SED*1_P_-SS-*T.r.eg2*-AD-*DIT*1_T_) + (pRS42-G-$\Delta$ + *SED*1_P_-SS-*T.e.cbh1*-AD-*DIT*1_T_) + (pRS42-G-ChXI + *SED*1_P_-SS-*A.a.bgl1*-AD-*DIT*1_T_) + (pRS42-G-$\Delta$ + *SED*1_P_-SS-*C.l.cbh2*-AD-*DIT*1_T_)	ER_V2	ER_WT transformed with CRISPR plasmids, pCas9-NAT and sgRNA plasmids (pRS42-G-ChX, pRS42-G-ChXI, and pRS42-G-$\Delta$, respectively) and the homology repair templates *T.r.eg2*, *A.a.bgl1*, *T.e.cbh1*, and *C.l.cbh2*, respectively, in successive rounds of transformation	This study
Reference strains (enzyme activity)			
*S. cerevisiae* Y294 + pRDH147*::fur1*	Y294_EG2	*S. cerevisiae* Y294 containing pRDH147 (*T.r.eg2* gene under control of *ENO*1_P_ and *ENO*1_T_), *FUR1* disrupted (*fur1::LEU2*)	Brevnova et al. (2011)
*S. cerevisiae* Y294 + pMI529:*:fur1*	Y294_CBH1	*S. cerevisiae* Y294 containing pMI529 (*T.e.cbh1* gene under control of *ENO*1_P_ and *ENO*1_T_), *FUR1* disrupted (*fur1::LEU2*)	Davison et al. (2016)
*S. cerevisiae* Y294 + ySFI*::fur1*	Y294_BGL1	*S. cerevisiae* Y294 containing ySFI (*S.f.bgl1* gene under control of *PGK*1_P_ and *PGK*1_T_), *FUR1* disrupted (*fur1::LEU2*)	Davison et al. (2016)

*Strains abbreviated as YI13_BECC, YI59_BECC, and MH1000_BECC in Minnaar and Den Haan (2023) will be referred to as YI13_V1, YI59_V1, and MH1000_V1, respectively, in this text. *SS* SED1 secretion signal, *AD* anchoring domain

Plasmids were propagated from *Escherichia coli* DH5$\alpha$ transformants maintained in 40% (v/v) glycerol stocks at −80 °C, by streaking cultures on LB agar (0.5% yeast extract, 1% tryptone, 1% NaCl, and 2% bacteriological agar) supplemented with 100 µg.mL^−1^ ampicillin (Roche, Basel, Switzerland), followed by overnight incubation at 37 °C (Supplementary Table S1). To prepare cultures for plasmid DNA isolation, single colonies were inoculated in liquid LB broth supplemented with 100 µg.mL^−1^ ampicillin, followed by overnight incubation at 37 °C on a rotary wheel.

### Plasmid DNA isolations, restriction digestion, and PCR amplification

Plasmid DNA isolation from *E. coli* DH5$\alpha$ cultures was performed using the ZymoPure Plasmid DNA Isolation kit (Zymo Research, Tustin, CA, USA), according to the manufacturer’s instructions. Quantitative analysis was then conducted using a NanoDrop 2000 (Thermo Scientific) to determine the concentration of isolated DNA. To verify the sizes of each respective gene cassette and/or CRISPR sgRNA sequence, isolated plasmid DNA was subjected to restriction digestion at 37 °C using *Pac*I, *Asc*I, *Eco*RI, *Xho*I, and/or *Hin*dIII (Thermo Fisher Scientific, Waltham, MA, USA), followed by separation on an 1% agarose gel. Following confirmation, gene cassettes were PCR amplified using Taq 2 × Master Mix RED (Ampliqon, Odense, Denmark) and specific primers to generate homology repair templates for use in electro-transformation of yeast strains (Supplementary Table S2).

To purify the resolved gene cassette PCR products and CRISPR plasmid DNA from the agarose gels, DNA bands were extracted using the freeze-and-squeeze method (Thuring et al. 1975), followed by phenol:chloroform:isoamyl-alcohol (PCI; 25:24:1) purification. Once purified, amplicons were subjected to dialysis against purified water on a 0.025-µm MCE membrane filter (Merck Millipore, Burlington, MA, USA) and subjected to quantitative spectrophotometric analysis using a NanoDrop 2000 to determine the DNA concentration and purity.

### Electro-transformation of yeast strains with CRISPR plasmids and a core set of cellulase encoding genes, screening of putative transformants

Through successive rounds of transformations, the core set of cellulase-encoding genes (*T.r.eg2*, *T.e.cbh1*, *C.l.cbh2*, *A.a.bgl1*) was integrated into the genomes of the parental strains, using different CRISPR sgRNA plasmids and the pCas9-NAT plasmid (Supplementary Table S1, Table 1). To achieve this, the lithium-acetate-based protocol described by Cho and co-workers (1999), with some minor adaptations (Moriguchi et al. 2016) was used. Briefly, harvested cells were washed with sterile distilled water, followed by resuspension in LiOAc/TE (0.1 M LiOAc, 10 mM Tris-HCl pH 8.0, and 1 mM EDTA). Resuspended cells were incubated at 30 °C for 45 min with shaking at 180 rpm, prior to the addition of 1 M dithiothreitol (DTT) for a further 15 min with shaking. Cells were then pelleted and washed, prior to resuspension in electroporation buffer (1 M sorbitol, 20 mM HEPES). Competent cells were transformed with ~10 µg homology template DNA and ~1 µg CRISPR plasmid DNA under standard electroporation conditions (1.4 kV, 200 ohms, 25 µF) using a MicroPulser (BioRad, Hercules, CA, USA). Following electroporation, cells were suspended in 1 mL YPD broth supplemented with 1 M sorbitol, followed by overnight incubation at 30 °C with shaking at 180 rpm. The transformation mixture was then plated on YPD agar media supplemented with 100 µg.mL^−1^ CloNAT and/or 200 µg.mL^−1^ Geneticin G418, as required, and incubated at 30 °C for 48–72 h.

Putative positive transformants obtained from the transformation plates were then screened for positive gene integrations. Briefly, colonies were subcultured onto fresh YPD agar media supplemented with 100 µg.mL^−1^ CloNAT and/or 200 µg.mL^−1^ Geneticin G418, followed by incubation at 30 °C for 48 h. A single colony for each streaked transformant was then inoculated in 5 mL YPD media and incubated overnight at 30 °C with shaking at 180 rpm. Quick yeast DNA isolation extractions were then performed as described by Hoffman and Winston (1987), and extracted yeast DNA was used as DNA templates to confirm the presence of integrated cellulase genes and/or correct target intergenic chromosomal regions with PCR analysis as described in Supplementary Table S2. Notable strains constructed in this study were submitted to the Biobanks South Africa Yeast Culture Collection at the Department of Microbiology and Biochemistry, University of the Free State. Strain collection numbers are detailed in the supplementary material (Supplementary Table S3).

### Enzymatic assays

To identify transformants with superior secretory profiles for all heterologous enzymes, preliminary activity assays were conducted by inoculating 5–10 PCR-confirmed positive transformants in 5 mL YPD broth for 48 h at 30 °C on an orbital shaker at 180 rpm. Superior cellulase secretors for each respective isolate were selected based on high individual enzyme secretory profiles, with the main determinant being CBH1 activity. Confirmed isolates were then cultivated in biological triplicates in 10 mL YP media supplemented with 2% (v/v) D-glucose under various process-relevant conditions, namely (1) optimal conditions 30 °C, (2) elevated temperatures 37 °C, and (3) in the presence of a weak acid (3 g.L^−1^ acetic acid at 30 °C), respectively, for 72 h with shaking at 180 rpm.

Endoglucanase II (EG2) activity was quantitated using the dinitrosalicylic acid (DNS) method as described by Bailey and co-workers (1992), using sodium acetate (50 mM, pH 5) as buffer and carboxymethyl cellulose (CMC, 1% w/v) as substrate as previously described (Minnaar and Den Haan 2023). Briefly, cell-free supernatants and/or whole cell cultures were incubated with the CMC substrate at 50 °C for 60 and/or 10 min, respectively, after which the reactions were inhibited with the addition of DNS. The volumetric values (U.L^−1^) obtained were normalized with the dry cell weight (DCW) of each respective isolate (Meinander et al. 1996), and the enzyme activities were expressed as units/gDCW. A DNS/glucose standard curve in the range of 0.25–10 g.L^−1^ was used for enzyme activity determinations. For all assays, one unit (U) was equivalent to the amount of enzyme required to release 1 µmol of reducing sugar or equivalent per minute.

Cellobiohydrolase I (CBH1) activity was quantitated using soluble fluorescent 4-methyllumberiferyl-$\beta$-lactopyranoside (MU-lac) (Sigma) as substrate, as described by Ilmén and co-workers (2011). Briefly, cell-free supernatants and/or whole cell cultures were incubated in 1:1 ratio with the substrate at 37 °C for 20 min, followed by inhibiting the enzyme reactions with 1 M Na_2_CO_3_ prior to measuring fluorescence (excitation wavelength = 355 nm; emission wavelength = 460 nm) using a FLUOstar Omega Microplate Reader (BMG LABTECH, Ortenberg, Germany). A methylumbelliferone MU standard curve was set up in the range of 0.63–20 µM and was used to compare against the amount of fluorescence emitted by each sample. Enzyme activity was expressed as mU/gDCW.

$\beta$-glucosidase I (BGL1) activity was quantitated using $\rho$-nitrophenyl-$\beta$-D-glucopyranoside (pNPG) as substrate, as described by Van Zyl and co-workers (2014). Briefly, whole cell cultures were assayed at 50 °C for 30 min, prior to inhibiting the enzyme reaction with 1 M Na_2_CO_3_ before spectrophotometric analysis of the supernatant at 400 nm. A pNP standard curve in the range of 0.075–1.25 mM was set up and used to compare against the volumetric values obtained for each respective sample. Enzyme activity was expressed as U/gDCW.

### Avicel conversion

To evaluate the efficacy of the rudimentary cellulase complex for Avicel hydrolysis, cell cultures were inoculated at a 1:1 ratio with a mixture of 2% (w/v) Avicel PH-101 (Fisher Scientific, Hampton, NH, USA), and sodium acetate (50 mM, pH 5.0), as described by Chetty and co-workers (2022). Avicel is a high-purity, fine powder composed of over 99% microcrystalline cellulose (MCC), specifically alpha cellulose. It is produced via acid hydrolysis of wood pulp and features particle sizes of ~20 µm (Avicel PH-105) or ~50 µm (Avicel PH-101) and a moisture content of under 5%. To inhibit uptake of reducing sugars by yeast cells, methylglyoxal (100 mM) (Sigma) was added to the reaction mixture. The reaction plate was then incubated at 35 °C with shaking at 1000 rpm in a Heidolph Titramax 1000 microplate shaker/incubator. Samples were extracted at 0, 24, and 48 h to measure the amount of glucose liberated by enzymatic hydrolysis, using an adapted DNS/glucose assay procedure (Den Haan et al. 2013; Minnaar and Den Haan 2023).

### Growth analysis

Cellular growth tests for wild-type and recombinant yeast cultures were conducted as described by Chetty and co-workers (2022). In 5 mL YP liquid media supplemented with 2% (w/v) D-glucose, yeast cultures were inoculated and incubated on an orbital shaker at 180 rpm, at 30 °C overnight. In 10 mL YP liquid media supplemented with 2% (w/v) D-glucose, overnight cultures were inoculated to an initial OD_600_ of 0.05, followed by incubation at 30 °C on an orbital shaker at 180 rpm. Growth was then monitored by extracting samples every 2 h until the stationary phase was reached. Appropriate dilutions were made at each sampling point, followed by measuring turbidity at 600 nm using a FLUOstar Omega Microplate Reader (BMG LABTECH, Ortenberg, Germany). Growth analyses were conducted in biological triplicates, and the OD values obtained were represented as the average of repeats against time, with their respective standard deviations.

### Strain robustness evaluations against bioethanol-related production and secretion stresses

Wild-type and recombinant strains were cultivated at 30 °C for 48 h in 10 mL YP liquid media supplemented with 2% (w/v) D-glucose, on an orbital shaker at 180 rpm. Cell densities were then measured at 600 nm for standardization, using YPD as diluent to attain an OD_600_ of 1.0 for each culture in a final volume of 1 mL. Ten-fold serial dilutions were then performed, followed by spotting 3 µL of each dilution on YPD agar media supplemented with the appropriate inhibitory component. The inhibitory components screened for which were specific to bioethanol production processes included ethanol (6% w/v), NaCl (1.2 M), and acetic acid (5 g.L^−1^). To evaluate strain robustness against heat stress, strains were cultivated on YPD agar at 30 °C (optimal) and 42 °C (supra-optimal). To evaluate strain robustness against secretion and cell wall stresses, strains were cultivated on YPD agar media supplemented with dithiothreitol (DTT, 20 mM) or Congo Red (CR, 800 µg.mL^−1^), respectively. All plate assays were conducted at 30 °C for 48 h, unless otherwise stated. Upon viewing plates, strains were compared with regard to their sensitivity towards various inhibitors or conditions based on their growth at various dilutions.

### Chitin determinations with Calcofluor White cell staining

Fresh single yeast colonies were inoculated in 10 mL YP liquid media supplemented with 2% (w/v) D-glucose for cultivation at 30 °C with shaking at 180 rpm. Samples were extracted every 24 h, after which cells were diluted to a final OD_600_ of 1.0. In 1 mL of cells, 10 µL of Calcofluor White stain and 10% KOH, respectively, were added. Cells were incubated at room temperature for 15 min, after which fluorescence (excitation wavelength = 355 nm; emission wavelength = 460 nm) was measured, using a FLUOstar Omega Microplate Reader (BMG LABTECH, Ortenberg, Germany). Data bars represent the mean of biological triplicates ± standard deviation, where means indicate the average fluorescence at a given timepoint.

### Transmission electron microscopy (TEM) to determine cell wall thickness

To determine the average thickness of yeast cell walls, fresh single yeast colonies were cultivated in 10 mL YP liquid media supplemented with 2% (w/v) D-glucose at 30 °C while shaking at 180 rpm. For microscopic analysis, cells were collected after 72 h to visualize the thickness of the cell walls once heterologous cellulases were produced. To view cells microscopically, collected cells were fixed in glutaraldehyde prepared in phosphate buffer (pH 7.2), followed by fixing in an osmium solution (OsO_4_). Once fixed, specimens were rinsed with water and dehydrated with ethanol before embedment into an Epon resin. Once embedded, cells were dissected with an ultramicrotome (LKB) and contrasted with uranyl acetate and lead acetate. Photographs were analyzed using ImageJ, and averages of cell wall thickness for cell populations per strain were calculated.

### Fermentation of microcrystalline cellulose

To test the strains’ capabilities as cellulosic ethanol producers, recombinant and wild-type strains were inoculated in YP liquid media supplemented with microcrystalline cellulose (Avicel PH-105, 2% w/v), as described by Chetty and co-workers (2022) with slight modifications. Briefly, V1 and V2 strains were inoculated from fresh overnight YPD cultures into 50 mL YP liquid media supplemented with 2% (w/v) D-glucose. To induce maximal cellulase production for inoculation of fermentation bottles, strains were incubated for 72 h at 30 °C, with shaking at 180 rpm. Additionally, isolates producing cellulases tethered to the yeast cell wall (V2 strains) were first pre-cultured in 10 mL YP liquid media supplemented with 2% (v/v) D-glucose at 30 °C overnight, followed by scaling up to a final volume of 400 mL YPD liquid media at 30 °C for 48 h, with shaking at 180 rpm. These cultures were then subjected to centrifugation at 1000×*g* for 10 min, after which cell pellets were washed multiple times with sterile distilled water.

Rubber-stoppered glass bottles (Lasec, Cape Town, South Africa) containing 10 mL double-strength YP liquid media (20 g.L^−1^ yeast extract, 40 g.L^−1^ peptone) supplemented with 40 g.L^−1^ Avicel PH-105 (to achieve a final concentration of 20 g.L^−1^ Avicel PH-105) after inoculation and minimal D-glucose (0.05% w/v) were inoculated with 10 mL media from YPD pre-cultures (OD_600_ of 1.0), or with washed cells to a final concentration of 150 g.L^−1^ wet cell weight. Bottles were then incubated at 37 °C for 96 h with shaking at 180 rpm. Oxygen-limited conditions were maintained by piercing rubber stoppers with 0.8 × 25-mm syringe filters plugged with cotton wool to act as CO_2_ outlets. Samples (1.5 mL volumes) were extracted at 0, 24, 72, and 96 h, followed by centrifugation at 13,000 rpm for 10 min, after which they were stored at −20 °C until further analysis. To determine the ethanol concentration at each timepoint, an ethanol kit (MegaZyme, Bray, Ireland) was used, following the AOAC method 2019.08 outlined by the manufacturer.

### Scanning electron microscopy (SEM)

The hydrolysis of Avicel PH-101 was visualized using scanning electron microscopy (SEM). Samples taken at the start and end of fermentations on Avicel PH-101 were filtered through 0.45 µm MCE membrane filters (Millipore, Massachusetts, USA) and dried overnight at 60 °C. Dried samples were then loaded on 12-mm SEM aluminium pin stubs and gold-coated (10 nm) using a Leica EM ACE200 sputter coater (Leica Microsystems, Germany). Imaging was performed with a Zeiss Merlin field emission SEM (Carl Zeiss Microscopy, Germany) with the following operating conditions: 2–3 kV accelerating voltage, 89–100 pA beam current, and image detection with InLens secondary electron (SE) and SE2 detectors.

### Statistical analysis

Significant differences between enzyme activities, growth data, ethanol quantification, and/or cell wall thickness attained were investigated using two-tailed *T*-tests, assuming unequal variance. A *p*-value lower than 0.05 was deemed significant. Calculations were performed using Microsoft Excel.

## Results

### Strain construction and pathway design

As previously reported, expression of cellulase-encoding genes in natural isolates of *S. cerevisiae* conferred cellulolytic capabilities to the constructed strains that were sufficient for the partial breakdown of microcrystalline cellulose (i.e. Avicel) (Minnaar and Den Haan 2023); however, the expression levels of the cellulases were low, negatively affecting the conversion of crystalline cellulose to ethanol. Thus, we aimed to enhance the cellulolytic capability of the natural isolates, YI13 and YI59, using a different design strategy for cellulase expression, namely tethering all cellulase enzymes to the cell wall. In addition, the *SED1* promoter and *DIT1* terminator were used to express all cellulase-encoding genes as these were previously shown to be superior to other promoters and terminators tested. The strain construction strategy and engineered metabolic pathway implemented in this study are illustrated in Supplementary Fig. S1 which depicts the cell surface-tethering approach employed for cellulase display, as well as the basic architecture of the gene cassettes used for strain engineering. In addition, the engineered metabolic module is presented, highlighting the heterologous expression of cellulases that enables the hydrolysis of crystalline cellulose into glucose, which can subsequently be assimilated and metabolized by the cell to support growth and ethanol production.

Successive rounds of CRISPR-Cas9-based transformations were used to integrate a core set of cellulase-encoding genes (*T.r.eg2*, *T.e.cbh1*, *C.l.cbh2*, *A.a.bgl1*) into the genomes of the parental strains as summarized in Table 1. Strains designated as “V1” had all cellulase-encoding genes expressed as free enzymes into the extracellular medium, except *A.a.bgl1* which was tethered to the cell surface. Conversely, “V2” designated strains had all cellulase-encoding genes tethered to their cell surfaces. Initial transformants were confirmed to contain all genes of interest with PCR, after which strains were screened for cellulase activities to account for clonal variation among transformants. CBH1 activity was used as the main determinant for selected strains due to the central role of this enzyme in the degradation of crystalline cellulose. The industrial strain Ethanol Red® (ER) was included as a benchmark due to its widespread application in established bioethanol production processes. In parallel, MH1000_V1, described in our previous study, was retained as a reference strain to ensure direct comparability with our previous results. Integration of cellulase-encoding genes at different chromosomal intergenic regions was confirmed to be successful, using PCR analysis (data not shown). Amplicons corresponding to 1372 bp, 1675 bp, 900 bp, and 602 bp were confirmative of successful integration for *T.r.eg2*, *T.e.cbh1*, *C.l.cbh2*, and *A.a.bgl1*, respectively. Once successful integration was confirmed, positive clones were subcultured on rich YPD medium without antibiotic selection to facilitate the loss of plasmids bearing the antibiotic-resistance markers. Subsequent recombinant clones were used for further analyses.

### Benchmarking recombinant cellulolytic natural isolates of* S. cerevisiae*

**Fig. 1:**
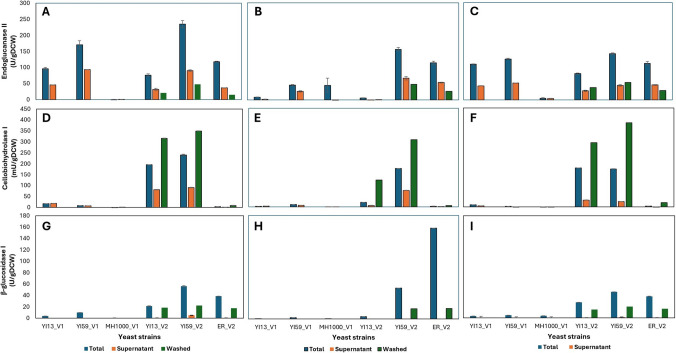
Individual cellulase enzyme activity evaluations of recombinant cellulolytic *S. cerevisiae* strains under different process-relevant conditions. Yeast strains were cultivated for 72 h in YP media supplemented with 2% (w/v) D-glucose with shaking at 180 rpm and incubated at **A**, **D**, and **G** control, optimal conditions of 30 °C; **B**, **E**, and **H** high temperature (37 °C); and **C**, **F**, and **I** optimal temperature of 30 °C in the presence of 3 g/L acetic acid. Volumetric values (U/L) obtained for individual culture fractions (blue bars, total cells; orange bars, supernatant; and green bars, washed cells) are standardized with the dry cell weight (DCW in g/L) of each strain, and the specific activities are expressed as (m)U/g DCW. Data bars represent the average of three biological repeats per strain, with error bars representing the mean ± standard deviation

Cellulase production of selected transformants was determined in the different strain backgrounds in various process-relevant conditions (Fig. 1). Wild-type strains yielded negligible enzyme activity, as was expected since these strains lack inherent cellulolytic capabilities (Supplementary Fig. S2). Natural isolate transformant *S. cerevisiae* YI59_V2 yielded superior secretory enzyme profiles for all individual enzymes assayed for, in all conditions tested, while MH1000_V1 yielded the poorest secretory enzyme profiles. The reference strain ER_V2, based on the industrial strain Ethanol Red®, yielded EG2 and BGL1 enzyme titres comparable to YI13_V2 and/or YI59_V2; however, negligible detectable CBH1 activity was produced by this strain.

Endoglucanase II (EG2) activity in the range of ~0–234.86 U/gDCW was obtained in the total cell fraction across all conditions tested, with YI59_V2 yielding superior activity in each of these conditions (234.86 U/gDCW at 30 °C, 156.71 U/gDCW at 37 °C, and 142.21 U/gDCW in the presence of 3 g/L acetic acid) (Fig. 1A, B, and C). Although markedly lower EG2 activity was measured in the supernatant fractions, the percentage of functional cellulases released into the medium by the free enzyme systems was notably higher than that of the cell-tethered systems. Additionally, the retention of EG2 activity on the cell wall of YI13_V2, YI59_V2, and ER_V2 was less than 50% across all conditions tested. Furthermore, although markedly lower than the superior secretor YI59_V2, YI59_V1 yielded 170.43 U/gDCW at 30 °C, outperforming the industrial strains ER_V2 and MH1000_V1, and both variants of natural isolate YI13. Interestingly, differences in EG2 secretory capacity were observed for all strain backgrounds across the different conditions, except for ER_V2, which yielded no difference in secretory capacity for EG2. Furthermore, YI13_V1 and YI13_V2 yielded secretory profiles that were similar across all the conditions, suggesting this strain background was not affected by the expression strategy used for EG2 expression.

Cellobiohydrolase I (CBH1) activity in the range of ~0–240.33 mU/gDCW was obtained for the total cell fraction across all conditions tested, with YI59_V2 once again displaying superior levels of activity (Fig. 1D, E, and F). Negligible detectable activity was obtained by all free enzyme–based systems, as well as the reference industrial strain ER_V2, across all conditions tested. Interestingly though, only the natural isolates YI59 and YI13 with the CBH1 attached to their cell walls yielded activity levels exceeding 150 mU/gDCW. Although these levels were relatively low compared to what is required for efficient enzymatic hydrolysis of feedstocks, the cell display strategy and genetic background of these strains clearly highlight the potential of enhancing CBH1 expression profiles. Furthermore, CBH1 levels in the presence of acetic acid yielded negligible differences between YI13_V2 (178.32 mU/gDCW) and YI59_V2 (173.78 mU/gDCW), while activity at high temperature by YI59_V2 (175.76 mU/gDCW) far exceeded that of YI13_V2 (20.24 mU/gDCW). An important phenomenon was observed when cells were washed prior to CBH1 activity assays, where CBH1 activity showed increases of more than 140% in the washed fractions compared to the total cell fractions in all conditions tested.

β-glucosidase I (BGL1) activity in the range of 0.16–158.02 U/gDCW was obtained across all conditions (Fig. 1G, H, and I), with ER_V2 yielding superior BGL1 activity at high temperature conditions (Fig. 1H). Nevertheless, YI59_V2 yielded consistent activity profiles across all conditions tested, with activity levels of 56.08 U/gDCW, 53.71 U/gDCW, and 46.13 U/gDCW at optimal temperature, high temperature, and in the presence of acetic acid, respectively. Conversely, the free enzyme–based systems yielded activity levels of ≤ 10 U/gDCW, despite identical strain backgrounds being used. It is important to note the retention efficiency of BGL1 on the cell wall of YI13_V2, which showed that more than 50% of the BGL1 was retained after washing. However, ER_V2 retained less than 45% of the BGL1 activity after washing.

### Avicel conversion efficacy

**Fig. 2:**
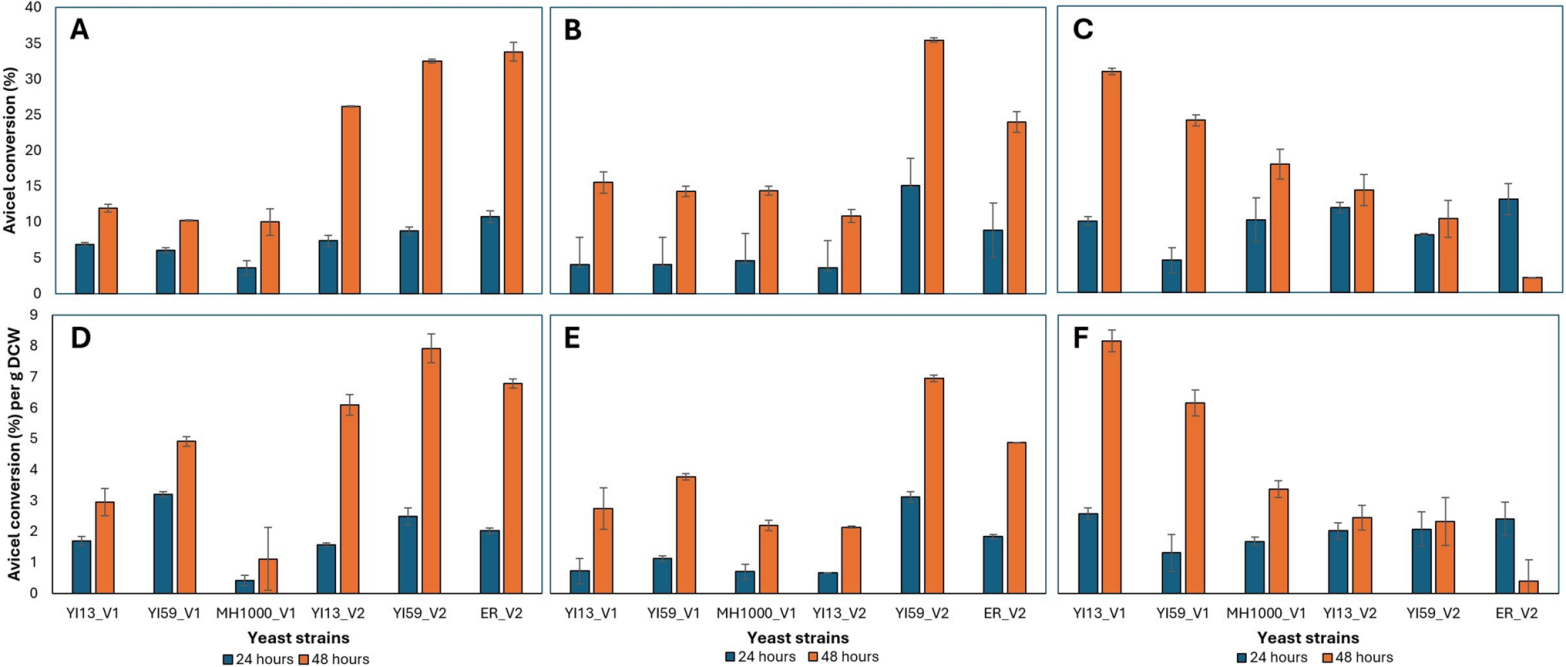
Avicel hydrolysis efficacy of recombinant strains in different process-relevant conditions. Yeast strains were cultivated in **A**, **D** optimal (i.e. 30 °C); **B**, **E** supra-optimal (i.e. 37 °C); and **C**, **F** acetic acid (3 g/L)-containing conditions for 72 h on an orbital shaker at 180 rpm. Total cell cultures were then inoculated at a 1:1 ratio in an Avicel-containing mixture, with the addition of methylglyoxal (100 mM) to suppress residual sugar uptake by cells, followed by incubation at 35 °C with shaking at 1000 rpm. Avicel hydrolysis (%) (**A**–**C**) was evaluated based on residual sugar determination and standardized with dry cell weight (DCW) (**D**–**F**). Data represent the average of three biological repeats per strain, with error bars representing the mean ± standard deviation

Avicel conversion efficacies appear to be strongly protein-specific, with distinct profiles exhibited by individual strain backgrounds in different process-relevant conditions (Fig. 2). While the cell-tethered-based systems yield conversion efficacies in the ranges of 26.15–33.79% and 10.87–35.44% at optimal (Fig. 2A) and high temperature (Fig. 2B) conditions after 48 h, respectively, the free enzyme–based systems were superior in the presence of acetic acid (Fig. 2C). Similar conversion efficacies were also detected among the free enzyme–based systems at optimal and high temperature conditions (Fig. 2A and B), although standardization of measurable conversion efficacies with individual strain background’s dry cell weights yielded more pronounced differences between strains (Fig. 2D and E). A striking result emerges with ER_V2 which yields an Avicel conversion efficiency comparable to that of YI59_V2 and YI59_V1 under high temperature conditions, despite this strain yielding negligible measurable CBH1 activity (Fig. 1B). Overall, the range of conversion efficacies detected between different process-relevant conditions was similar; nevertheless, differences were detectable between the expression strategies used for cellulase expression as well as between different strain backgrounds.

### Growth kinetics

**Fig. 3:**
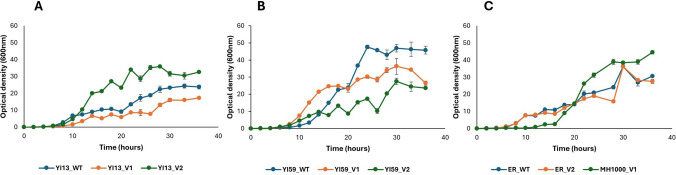
Growth kinetics of recombinant yeasts (V1 and V2) compared to their wild-type parental strains. Yeast isolates **A** YI13, **B** YI59 and **C** Ethanol Red and MH1000 were cultivated in optimal cultivation conditions (30 °C) for the duration of the growth analysis. Samples were extracted at 2-h intervals, and appropriately diluted prior to measuring optical density at 600 nm. Data points represent the average of three biological repeats per respective strain isolate, and error bars indicate mean ± standard deviation

To evaluate how the growth kinetics of individual strain backgrounds was affected when cellulases were expressed using different strategies, cellular growth was monitored over the course of 40 h in YPD liquid media (Fig. 3 and Table 2).

**Table 2:** Growth kinetics of recombinant yeasts compared to their wild-type parental strain

Yeast strains	Specific growth rate, µ (h^−1^)	Doubling time (hrs)
YI13_WT	0.54	1.28
YI13_V1	0.37	1.86
YI13_V2	0.43	1.62
YI59_WT	0.37	1.89
YI59_V1	0.49	1.43
YI59_V2	0.29	2.40
ER_WT	0.39	1.79
ER_V2	0.32	2.19
MH1000_V1	0.33	2.08

While the wild-type strains (YI13, YI59, and ER) displayed higher specific growth rates than their engineered counterparts, the observed differences among engineered strains were strongly influenced by the particular cellulase expression strategy employed in each strain background. YI13_V2 exhibited a lower specific growth rate than YI13_V1. Conversely, YI59_V2 yielded a slower growth rate than the wild type, while YI59_V1 yielded a faster specific growth rate than the wild type. Similarly, the engineered ER strain also yielded a lower specific growth rate than the wild-type counterpart.

### Strain robustness

**Fig. 4:**
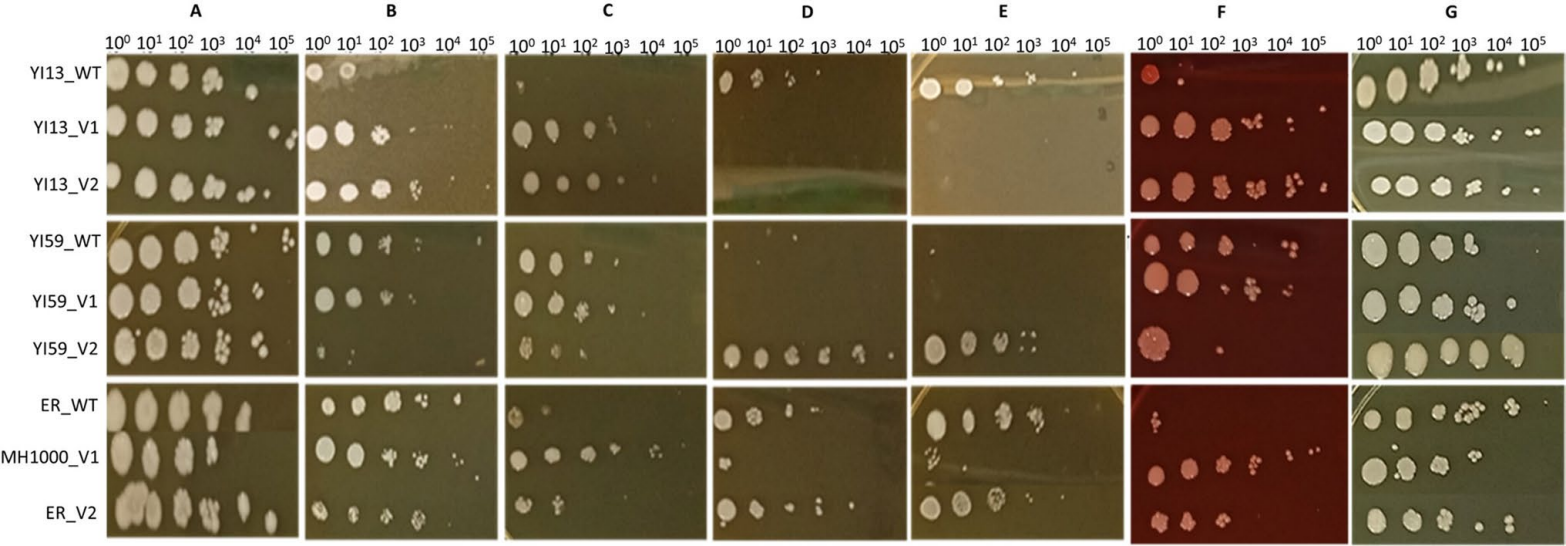
Strain robustness evaluations under different bioprocess-relevant conditions. Yeast strains were cultivated in YP liquid broth supplemented with 2% (w/v) D-glucose for 48 h at 30 °C on a rotary shaker at 180 rpm. Ten-fold serial dilutions starting from an initial OD_600_ of 1 were then spotted on YPD agar supplemented with appropriate inhibitors commonly associated with bioprocesses, prior to incubation at 30 °C, or unless otherwise stated, for 48 h. **A** Control, with no inhibitor present; **B** high temperature at 42 °C; **C** osmo-tolerance, 1.2 M NaCl; **D** weak acid stress, 5 g/L acetic acid; **E** ethanol tolerance, 6% (w/v); **F** cell wall stress - Congo Red (800 µg.mL.^−1^); and **G** secretion stress - 20 mM dithiothreitol (DTT)

An important indicator for strain suitability for cellulosic bioethanol production is strain robustness against the myriads of stresses present in general fermentation environments. To investigate the suitability of the strains in this study, spot plate assays using different inhibitory conditions were employed (Fig. 4).

Based on the dilutions per isolate plated, growth at optimal temperature (30 °C; control) is maximal, illustrating the proficiency of these strains under optimal growth conditions (Fig. 4A). However, at high temperatures, growth defects are observed (Fig. 4B). Low to moderate growth was exhibited by YI13 and YI59 strains, for both wild-type and engineered variants. As expected, the ER and MH1000 strains showed maximal growth, as inferred by their industrial applicability. Similar sensitivity profiles are also detected in the presence of high salt concentrations (1.2 M NaCl) (Fig. 4C), where both YI13 and YI59 engineered strains yielded moderate growth, while YI13_WT and ER_WT showed sensitivity to growth in this condition. An interesting result was obtained by YI59_V2 in 6% (v/v) ethanol and in the presence of 5 g/L acetic acid, where moderate to high tolerance was exhibited, respectively, compared to YI59_WT and YI59_V1 which exhibited extreme sensitivity (Fig. 4D and E). A similar result was also obtained by the reference industrial strains MH1000 and ER.

As a result of rational engineering done to the wild-type strains, strain variants were also evaluated for their sensitivity towards cell wall and secretion stresses (Fig. 4). For both YI13 and ER wild types, sensitivity to the cell wall stressor Congo Red appears to be prevalent, while the YI59_WT strain exhibits moderate growth in the presence of this stressor (Fig. 4F). Similarly, the wild-type strain for YI59 also exhibits moderate sensitivity towards secretion stress, although the engineered variants of this strain background show remarkable tolerance (Fig. 4G). A striking result, however, was the incidence of high tolerance exhibited by YI13_V1, YI13_V2, YI59_V1, and MH_V1 against cell wall stress, while extreme sensitivity was detected for YI59_V2 (Fig. 4F). Based on all the above results, it became apparent that the YI59 strain background was the most affected by the expression strategy used for cellulase expression, as indicated by the superiority in individual enzyme expression, Avicel conversion assays, growth kinetics, and strain robustness against various process-relevant and cell wall and secretion stresses. For this reason, the remaining experimental procedures were only conducted on the YI59 strain variants.

### Cell wall compositional analysis using transmission electron microscopy (TEM) and fluorescence

**Fig. 5:**
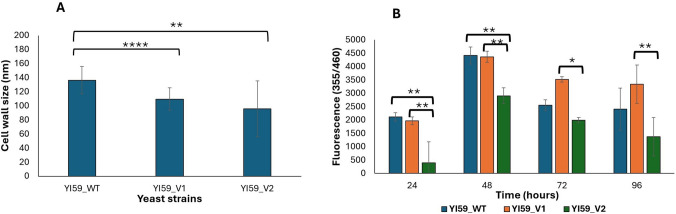
Cell wall size and chitin measurements of YI59-based strains. Yeast strains were cultivated in YP media supplemented with 2% (w/v) D-glucose to determine the **A** thickness of cell walls with transmission electron microscopy (TEM) and/or to **B** measure the fluorescence emitted by Calcofluor White bound to chitin in yeast cell walls over the course of 96 h. Data bars represent the mean of triplicate repeats per strain ± standard deviation. Statistical analysis was carried out using Student’s *T*-test (two-tailed assuming unequal variance where *P < 0.05, **P < 0.01, ****P < 0.00001)

To evaluate whether the cellulases anchored to the cell wall might have influenced the distribution of cell wall components, natural isolate YI59_WT and its two engineered variants, YI59_V1 and YI59_V2 were subjected to TEM (Fig. S3A-C and Fig. 5A) and fluorescence analysis after Calcofluor White staining (Fig. 5B).

Using a 72-h cell culture, yeast cells were analyzed to measure the average width of the yeast’s cell walls. As depicted on a 500-nm scale, cell wall thickness was measured using ImageJ analysis software, followed by calculating the average cell and cell wall sizes for multiple cells in the population. While estimation of cell wall thickness is impossible with the naked eye, cells of uniform conformation with little to no fuzzy edges were selected for analysis. As illustrated in Supplementary Fig. S3A–C, organelles and internal components consistent with mature cells were present, and no buds were detected that may have influenced the thickness at any point on the cell wall. From these microscopic images, cell wall size was determined for 14 to 20 cells each of different strains and averages were calculated (Fig. 5A). Cell wall thickness (nm) drastically decreased in the YI59_V2 strains compared to the wild type and free enzyme–based systems, confirming a structural change in cell wall organization because of the tethering of heterologous enzymes. To further strengthen this observation, the lower fluorescence detected by YI59_V2 with Calcofluor White (Fig. 5B) directly reflected the reduced amount of chitin, a critical structural polysaccharide in the cell wall. Furthermore, over the course of the 96 h in which fluorescence was measured, a drastic decrease emerged, likely pointing to a weakening of the cell wall over time.

### Fermentative conversion of Avicel to ethanol

**Fig. 6:**
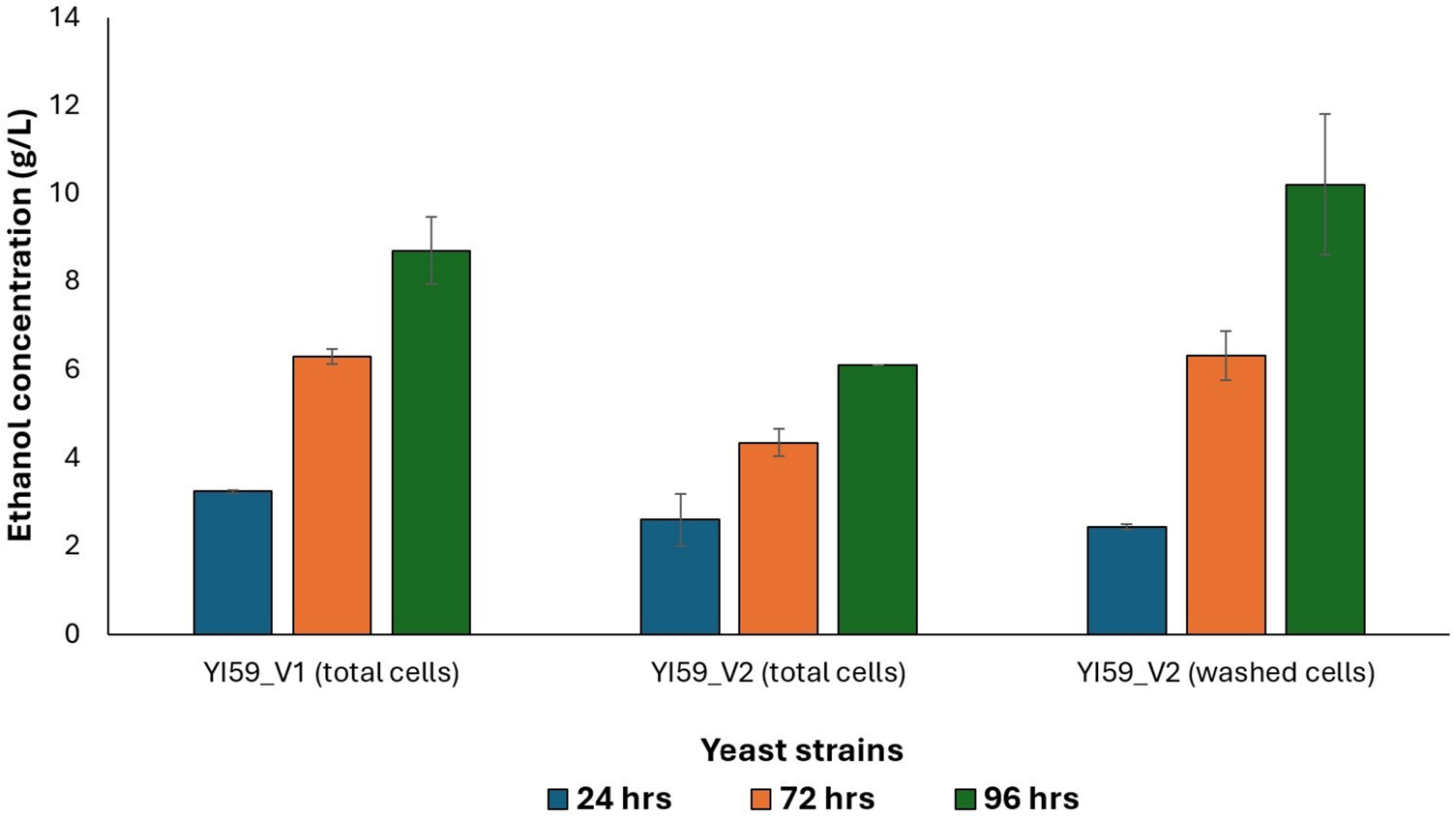
CBP fermentations on Avicel PH-105. Pre-inoculums of YI59-based strains were prepared in YP media supplemented with 2% (w/v) D-glucose for 72 h with shaking at 180 rpm. Rubber-stoppered fermentation bottles were inoculated with total cells (10 mL) and washed cells (150 g wet cell weight) with the addition of glucose (0.5 g/L), for fermentation of 20 g/L Avicel PH-105 over the course of 96 h. Samples were collected at 0, 24, 72, and 96 h, followed by ethanol quantification. Data bars represent the mean of triplicates ± standard deviation

To evaluate how our newly constructed strains YI59_V1 and YI59_V2 would perform on a recalcitrant substrate, we attempted to ferment microcrystalline cellulose (Avicel PH-105) for a duration of 96 h at 37 °C (Fig. 6). An ethanol concentration of 10.2 g/L was achieved by YI59_V2 (washed cells) at 96 h, while YI59_V1 (total cells) reached 8.7 g/L. Evaluation of process performance exhibited by YI59_V2 yielded ethanol productivity and ethanol yield corresponding to 0.106 g/L.h^−1^ and 0.449 g/g substrate, respectively. A faster ethanol production rate was achieved by the YI59_V2 washed cells, where 6.3 g/L was obtained at 72 h, while the YI59_V2 (total cells) managed to achieve a similar concentration at 96 h (6.1 g/L). Note that the latter fermentation had a significantly lower cell load at the point of inoculation. A steady increase in ethanol concentrations was exhibited by all strains up to 96 h.

**Fig. 7:**
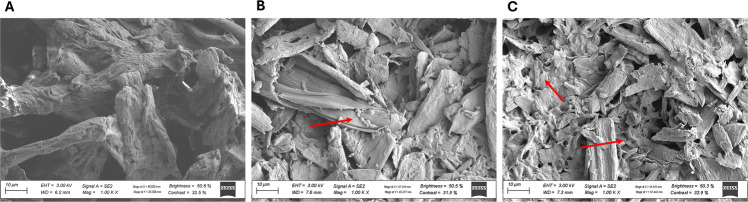
Scanning electron microscopy (SEM) analysis of fermentations conducted by YI59 variants on Avicel PH-101. **A** The control consisting of only an Avicel-YP mixture and no fermenting inoculum. Sample aliquots of day 7 fermentations were subjected to SEM analysis to evaluate the hydrolysis efficiency of cellulases expressed in *S. cerevisiae*: **B** YI59_V1 and **C** YI59_V2

To visualize hydrolysis efficiency and gain insight into the potential synergistic activity of the rudimentary cellulase complex acting on microcrystalline cellulose, scanning electron microscopy was employed as a qualitative assessment tool (Fig. 7). Large fragments of cellulose fibrils detected in Fig. 7A at 0 h reflect the recalcitrant nature of the fermentation substrate used here, Avicel PH-101. Saccharification, hydrolysis and fermentation with YI59_V1 and YI59_V2 clearly lead to breakdown of the cellulose fibrils into smaller fragments, with the YI59_V2 (Fig. 7C) displaying greater degradation than that of YI59_V1 (Fig. 7B). As cellulases are attached to the cell wall of the YI59_V2 strain, cells were localized in closer proximity to the cellulose fibrils (red arrows), while the cells in the YI59_V1 culture were more scattered and less dense.

## Discussion

In this study, successful enhancement of cellulase expression levels was proven to be not only distinctly host or protein specific, but also largely dependent on the design strategy used (Fig. 1). Evidence of this was exemplified by the expression profiles exhibited by the cell-tethered systems, where YI59_V2 was the superior secretor for all individual enzymes, under all process-relevant conditions tested, compared to the free enzyme systems. Based on extensive research led by Inokuma and co-workers, the use of stress-induced *SED1*_P_ and *DIT1*_T_, the Sed1 signal peptide, in combination with the glycosylphosphatidylinositol (GPI) anchorage systems, led to greatly enhanced expression profiles of EG2, CBH1, and BGL1, individually and/or in multi-gene combinations, in the laboratory strain *S. cerevisiae* BY4741 (Inokuma et al. 2014, 2016, 2021). Recently, expression profiles of xylanase and xylosidase under transcriptional control of *TDH3*_p_, *SED1*_p_, and *ENO1*_P_ was tested and shown to yield greatly enhanced levels under control of *TDH3*_P_ and *SED1*_P_ compared to *ENO1*_P_ (Fortuin and Den Haan 2025). These findings align with the present study, in which cellulase-encoding genes expressed under the *SED1*_P_/*DIT1*_T_ cell-tethered system outperformed those expressed under the *ENO1*_P/T_ or *PGK1*_P/T_ free enzyme systems. While a range of secretory capacities was observed for individual cellulase enzymes between different strain backgrounds, natural isolates outperformed the reference industrial strains. Although limited quantitative data is available on these natural isolates, their improved secretion phenotypes likely suggest an interplay between the protein secretory pathway and general environmental stress response (Davison et al. 2019; 2020; DeWitt et al. 2019). Furthermore, natural adaptation in harsh environmental conditions likely contributed to a greater genetic complexity, variation, and robustness as described previously (Davison et al. 2016; Steensels et al. 2014).

Furthermore, we showed that the expression of individual cellulases was not directly proportional to the Avicel conversion efficacy of the rudimentary cellulase complex (Fig. 2). While the secretory capacity for individual enzymes in YI59_V1 was markedly lower than YI59_V2 in acetic acid-stressed conditions, a 2.32-fold higher efficacy was obtained by YI59_V1. Similarly, extremely low CBH1 and BGL1 activity levels were obtained by YI13_V1, yet its conversion efficiency was 1.28-fold and 2.99-fold higher than YI59_V1 and YI59_V2, respectively. We therefore postulate that the higher efficacy exhibited by the free enzyme systems was likely attributed to the ability of BGL1 to use both cellodextrins and cellobiose as substrates. As such an enhanced synergy likely existed between EG2 and BGL1, which in turn enhanced CBHs’ catalytic efficiency. Additionally, these results clearly illustrated that secretory profiles obtained from enzyme assays utilizing soluble substrate analogues are not directly proportional to the processive behaviour of a cohort of cellulases hydrolyzing insoluble substrates (Sørensen et al. 2015). It is also important to note that CBH2 activity levels could not be quantitated due to unavailability of an appropriate soluble substrate; thus, its contribution to the synergistic action of the cellulase complex could not be determined. Furthermore, no gene copy number or transcriptomic analysis was conducted on any of the engineered strains; hence, no inferences can be drawn about optimal ratios of cellulase production in the complex, though the relevance of these indirect metrics for estimating enzyme abundance is itself debatable.

Evidently, the design strategy used for heterologous protein affected the growth kinetics of the strains, as reduced specific growth rates in the engineered strains resulted in longer doubling times. A significantly reduced specific growth rate and lower final biomass yield was observed for YI59_V2, in comparison with YI59_WT (0.37 h^−1^) and YI59_V1 (0.49 h^−1^). This was expected, as elevated growth rates in the early exponential phase would have resulted in a faster exhaustion of nutrients, subsequently leading to the upregulation in carbohydrate metabolism–related stress response genes (Brauer et al. 2008) and ultimately upregulation in cellulase-encoding genes (Liu et al. 2013; Rebnegger et al. 2014). In doing so, cells would have experienced increased heterologous protein production at the expense of biomass production, as increased amounts of the available intracellular resources were redirected to the protein secretory pathway instead of being used for normal cellular growth (Huang et al. 2017). Furthermore, the lower biomass production and/or slower doubling time experienced by the cell-tethered strains is likely explained by the metabolic burden imposed on cells due to cell tethering (Ding et al. 2018).

Interestingly, we also demonstrated that the design strategy used for cellulase expression had a notable impact on overall strain robustness against a multitude of stresses. The recombinant strains YI13_V1, YI13_V2, and YI59_V1 showed stress profiles that demonstrated moderate to high tolerance against heat, osmotic, cell wall, and secretion stress; however, sensitivity was exhibited to ethanol (6% w/v) and acetic acid (5 g/L) (Fig. 4D and E). Surprisingly though, YI59_V2 exhibited an opposite tolerance profile. Enhanced tolerance exhibited towards ethanol and acetic acid was balanced with sensitivity towards heat stress and cell wall stress. This latter result suggests that the yeast cell might have a thinner β-glucan and chitin layer, potentially caused by interference of the cellulases’ anchoring domains with the normal distribution of the cell wall components (Ribeiro et al. 2022). Thus, we postulate that increased susceptibility to high temperature was likely directly related to a potentially impaired cell wall biogenesis, as indicated by sensitivity to cell wall stress (Levin 2011). As a result, thinner cell walls increased the likelihood of taking up metabolites from the environment intracellularly, such as sugars/salt, ethanol, and acetic acid. Although this may result in the induction of cell death, the presence of cellulases on the cell surface of cells likely slowed this process.

To date, no yeast strains have been shown to produce heterologous proteins in optimal ratios at levels necessary for complete enzymatic saccharification, hydrolysis, and fermentation of cellulosic substrates to yield ethanol, in a one-pot configuration without exogenous cellulase cocktails. Nevertheless, extensive research into 2G bioethanol production has shown excellent progress in developing fit-for-purpose strains which was recently reviewed (Fortuin et al. 2025). In this study, the YI59_V2 (washed cells) fermentation of Avicel PH-105 achieved an ethanol concentration of 10.2 g/L, corresponding to 88% of the theoretical maximum ethanol yield. Furthermore, clear hydrolysis in cellulose fibrils was evident at the end of fermentation (SEM data), illustrating the capacity of YI59_V2 to saccharify, hydrolyze, and ferment Avicel microcrystalline cellulose into ethanol. Our findings compare favourably with a study conducted by Song and co-workers, who performed co-fermentation of corncobs with a consortium of *S. cerevisiae* strains expressing the core set of cellulases individually per strain (Song et al. 2016). After 96 h, an ethanol concentration of 6.37 g/L was obtained, which demonstrated the importance of synergism among the different cellulases. In another study, Singh and co-workers demonstrated a 1.7 g/L ethanol titre from 20 g/L crystalline cellulose after 96 h with the thermophilic *Clostridium* sp. strain (Singh et al. 2017). Similarly, Dong and co-workers reported a 5.1 g/L ethanol titre from the direct conversion of amorphous carboxymethyl cellulose, with the use of a recombinant *S. cerevisiae* strain engineered with EG, CBH, and BGL contained in a mini-cellulosome (Dong et al. 2020). By combining the individual cellulases required for the successful enzymatic hydrolysis of feedstocks, it became clear that an enhancement in synergy between the cellulases was achieved, ultimately leading to an enhancement in the ethanol titres obtained. This was also clearly illustrated by Liu and co-workers, who showed that cell-tethered systems yielded higher ethanol titres; however, compared to their secretion-based strains, limited differences in their conversion efficacies were observed (i.e. 27% and 32% for secretion and cell-tethered systems, respectively, from 10 g/L Avicel without the addition of exogenous cellulase cocktails) (Liu et al. 2016). Further optimization of the superior cell-tethered strain, by tweaking the optimal ratios of EG2:CBH1:CBH2:BGL1, yielded 2.9 g/L ethanol from 10 g/L Avicel which corresponded to 57% of the theoretical maximum ethanol produced (Liu et al. 2017). Based on these comprehensive studies, the ethanol titres obtained in our study compared favourably, in showing that cell-tethered systems yielded higher ethanol titres than the secretion-based strains, from the same strain backgrounds. Furthermore, supplementation of the Avicel medium with a minor amount of exogenous glucose (0.5 g/L) to stimulate cellular metabolism showed enhanced ethanol titres in the secretion-based strains in this study compared to previously reported results (Minnaar and Den Haan 2023). Building on these previous reports, our findings further demonstrate the potential of these engineered natural *S. cerevisiae* isolates as promising chassis organisms for cellulosic bioethanol production.

In this study, different expression strategies were used to construct cellulase-producing strains that were capable of improved cellulosic bioethanol. Among the strains evaluated through rational engineering, the YI59 background emerged as the most effective, outperforming others in cell-tethered enzyme production and Avicel conversion, while also displaying increased tolerance to diverse stress conditions. Cellulosic ethanol produced by YI59_V2 (washed cells) reached ~10 g/L after 96 h, which corresponded to ~88% of the theoretical maximum ethanol yield. This superior phenotype displayed by YI59_V2 demonstrates an intricate rewiring of regulatory networks as cells adapt to their environments, displaying the superiority of its genetic diversity. Therefore, a clear demonstration is given that the strain is well suited for one-pot bioconversion of microcrystalline cellulose, eliminating the requirement for external cellulase supplementation. Future studies will extend this work by evaluating the performance of the best-performing strain in fermentations using pretreated lignocellulosic hydrolysates, with the aim of assessing its robustness and productivity under industrially relevant conditions with higher substrate loadings. These investigations will include different process configurations, such as fed-batch fermentations, simultaneous saccharification and fermentation (SSF), and CBP setups.

## Supplementary Information

Below is the link to the electronic supplementary material.

ESM 1 PDF (503 KB)

## Data Availability

Data will be made available on request.
