# Supplementary material for: Engineering natural Saccharomyces cerevisiae isolates for enhanced one-step cellulosic ethanol production
Source: Appl Microbiol Biotechnol. 2026 May 8;110:198. doi: 10.1007/s00253-026-13830-0 (PMC13323205; doi:10.1007/s00253-026-13830-0)
Supplement: Supplementary file 1 [file 253_2026_13830_MOESM1_ESM.pdf]

Supplementary material for:

Engineering natural *S. cerevisiae* isolates for enhanced one-step cellulosic ethanol production

Letitia Sabina Minnaar [a], Kentaro Inokuma [b], Tomohisa Hasunuma [b, c], and Riaan den Haan* [a, d]

[a] Institute for Microbial Biotechnology and Metagenomics, Department of Biotechnology, University of the Western Cape, Bellville, 7530, South Africa

[b] Graduate School of Science, Technology and Innovation, Kobe University, 1-1 Rokkodai-cho, Nada-ku, Kobe 657-8501, Japan

[c] Engineering Biology Research Center, Kobe University, 1-1 Rokkodai-cho, Nada-ku, Kobe 657-8501, Japan

[d] Department of Microbiology, Stellenbosch University, Stellenbosch, 7600, South Africa

*Corresponding author

Table S1 Plasmids used in this study

Plasmid	Description and application	Reference
**pRDH180**	Contains *ENO*1$_P$ and *ENO*1$_T$, and *T.r.eg2* gene cassette (used to produce homology repair template DNA for *T.r.eg2* integration to be expressed as secreted free enzyme)	Brevnova et al. (2011)
**pMI529**	Contains *ENO*1$_P$ and *ENO*1$_T$, and *T.e.cbh1* gene cassette (used to produce homology repair template DNA for *T.e.cbh1* integration to be expressed as secreted free enzyme)	Ilmén et al. (2011)
**pMU784**	Contains *PGK*1$_P$ and *PGK*1$_T$, and *C.l.cbh2* gene cassette (used to produce homology repair template DNA for *C.l.cbh2* integration to be expressed as secreted free enzyme)	Ilmén et al. (2011)
**pIBG-SSAD**	Contains *SED*1$_P$ and *DIT*1$_T$, and *A.a.bgl1* gene cassette (used to produce homology repair template DNA for *A.a.bgl1* integration to be expressed as cell-tethered enzyme)	Inokuma et al. (2016)
**pIAUR-ClCBH2b-SSSD**	Contains *SED*1$_P$ and *DIT*1$_T$, and *C.l.cbh2* gene cassette (used to produce homology repair template DNA for *C.l.cbh2* integration to be expressed as cell-tethered enzyme)	Inokuma et al. (2021)
**pIL2EG-SSSD**	Contains *SED*1$_P$ and *DIT*1$_T$, and *T.r.eg2* gene cassette (used to produce homology repair template DNA for *T.r.eg2* integration to be expressed as cell-tethered enzyme)	Inokuma et al. (2016)
**pIU5-TeCBH1c-SSSD**	Contains *SED*1$_P$ and *DIT*1$_T$, and *T.e.cbh1* gene cassette (used to produce homology repair template DNA for *T.e.cbh1* integration to be expressed as cell-tethered enzyme)	Inokuma et al. (2021)
**pCas9-NAT**	Yeast episomal plasmid with *cas9* expression cassette	ADDGENE
**pRS42-G-ChX**	sgRNA scaffold plasmid that targets an intergenic region on Chromosome 10	Jacob et al. (2022)
**pRS42-G-ChXI**	sgRNA scaffold plasmid that targets an intergenic region on Chromosome 11	Kruger and Den Haan (2022)
**pRS42-G-ChXII**	sgRNA scaffold plasmid that targets an intergenic region on Chromosome 12	Jacob et al. (2022)
**pRS42-G-Δ**	sgRNA scaffold plasmid that targets delta sequences within the yeast genome	Jacob et al. (2022)
**pRS42-G-CAN1**	sgRNA scaffold plasmid that targets intergenic regions at *CAN1* sites	This laboratory

Table S2 Primers used for homology template and gene integration confirmations

Primer name	Sequence (5'-3')	Application
**Homology repair template amplification**		
**Ch10.ENO1$_P$-L**	GCAGTTATCTCTGTGTGYCCAGATCCCTT	Amplify homology repair template DNA (*ENO*1$_P$-*T.r.eg2*-*ENO*1$_T$) with Chromosome 10 target homology
**Ch10.ENO1$_T$-R**	CTACATGTAATTGTGCGGTGCAGGGAGG	
**DELTA-ENO1-L**	CTTAAGATGCTCTTCTTATTCTATTAAAAA	Amplify homology repair template DNA (*ENO*1$_P$-*T.e.cbh1*-*ENO*1$_T$) with delta (Δ) target homology
**DELTA-ENO1-R**	TAGAAAATGACTTCTAGGCGGGTTATCTACTG	
**DELTA-PGK1p-L**	CTTAAGATGCTCTTCTTATTCTATTAAAAATAGAAAAT GATCCCTCCTTCTTGAATTG	Amplify homology repair template DNA (*PGK*1$_P$-*C.l.cbh2*-*PGK*1$_T$) with delta (Δ) target homology
**DELTA-PGK1t-R**	GTTTGTTTGCGAAACCCTATGCTCTGTTGTTCGGATT TGAAACGCAGAATTTTCGAG	
**Ch11_SEDp-L**	TGTAAAACAGGTATTGGCTGCTTCATAGTACACCCAA	Amplify homology repair template DNA (*SED*1$_P$-SS-*A.a.bgl1*-AD-*DIT*1$_T$) with Chromosome 11 target homology
**Ch11_DITt-R**	TTGATTGGATATAGAAAATTAACGTAAGGCAGTATC GCAACTCTGAAATGTCAAAACGGTCGTCGTATAAATA AATGTTACTCCGCAACGCTTTTCTG	
**ChX_SEDp_L**	GCAGTTATCTCTGTGTCCAGATCCCTTTGAAGTAAAG	Amplify homology repair template DNA (*SED*1$_P$-SS-*T.r.eg2*-AD-*DIT*1$_T$) with chromosome 10 target homology
**ChX_DITt_R**	TTTATTGGATATAGAAAATTAACGTAAGGCAGTATC CTACAGTAATTGTGCGGTGCAGGGAGGCAATGTTTA GTGCTTACTCCGCAACGCTTTTCTG	
**DELTA_SEDp_L**	CTTAAGATGCTCTTCTTATTCTATTAAAAATAGAAAAT GAATTGGATATAGAAAATTAACGTAAGGCAGTATC	Amplify homology repair template DNA (*SED*1$_P$-SS-*T.e.cbh1*-AD-*DIT*1$_T$) with delta sequence target homology
**DELTA_DITt_R**	GTTTGTTTGCGAAACCCTATGCTCTGTTGTTCGGATT TGATTACTCCGCAACGCTTTTCTG	
**DELTA_SEDp_L**	CTTAAGATGCTCTTCTTATTCTATTAAAAATAGAAAAT GAATTGGATATAGAAAATTAACGTAAGGCAGTATC	Amplify homology repair template DNA (*SED*1$_P$-SS-*C.l.cbh2*-AD-*DIT*1$_T$) with delta sequence target homology
**DELTA_DITt_R**	GTTTGTTTGCGAAACCCTATGCTCTGTTGTTCGGATT TGATTACTCCGCAACGCTTTTCTG	
**Ch12_SED1p_L**	GCGTCCTACAGCGTGATGAAAATTTCGCCTGCTGCA	Amplify homology repair template DNA (*SED*1$_P$-SS-*C.l.cbh2*-AD-*DIT*1$_T$) with chromosome 12 target homology
**Ch12_DIT1t_R**	AGATATTGGATATAGAAAATTAACGTAAGGCAGTATC CTGTCAAACTTCTGAGTTGCCGCTGATGTGACACTGT GACTTACTCCGCAACGCTTTTCTG	
**CAN1_SEDp_L**	GGTCAATACCATTGAAAGATGAGAAAAGTAAAGAAT	Amplify homology repair template DNA (*SED*1$_P$-SS-*C.l.cbh2*-AD-*DIT*1$_T$) with CAN1 site target homology
**CAN1_DITt_R**	TGTAATTGGATATAGAAAATTAACGTAAGGCAGTATC GGCAATCATACCAATATGTCTTTGCTTAAGCTCTCTCT TCTTACTCCGCAACGCTTTTCTG	
**Confirmation of gene integrations**		
**ENO1_L**	GTAACATCTCTCTTGTAATCCCTTATTCCTTCTAGC	Confirm integration of *ENO*1$_P$-*T.r.eg2*-*ENO*1$_T$ in transformants
**EGR-Rev**	ATCTGGATTAGTAACTTGAGACAAAGCAG	
**ENO1_L**	GTAACATCTCTCTTGTAATCCCTTATTCCTTCTAGC	Confirm integration of *ENO*1$_P$-*T.e.cbh1*-*ENO*1$_T$ in transformants
**CBH1R_Rev**	TGTTGAGAGAAGTCGTCGGTGTCAC	

**SED1p_check-L**	GACAAGCAAAATAAAATACGTTCGCTC	Confirm integration of *SED*1$_{P}$-
**AaBGL_check_R**	GCTTGGCTGTCGCCACG	*A.a.bgl1-DIT1*$_{T}$ in transformants
**C.l.cbh2_L**	AGTCTTAATTAAACAATGGCCAAGAAGTTGTTC	Confirm integration of *PGK*1$_{P}$-
**C.l.cbh2_R**	AGTCGGCGCGCCTTAGAATGGTG	*C.l.cbh2-PGK*1$_{T}$ in transformants
**SED1p_check-L**	GACAAGCAAAATAAAATACGTTCGCTC	Confirm integration of *SED*1$_{P}$-SS-
**SED1anchor-R**	GGAAGAAGCAGATGTACTGTTGG	*T.r.eg2*-AD-*DIT*1$_{T}$ in transformants
**SED1p_check-L**	GACAAGCAAAATAAAATACGTTCGCTC	Confirm integration of *SED*1$_{P}$-SS-
**CBH1R-Rev**	TGTTGAGAGAAGTCGTCGGTGTCAC	*T.e.cbh1*-AD-*DIT*1$_{T}$ in transformants
**SED1p_check-L**	GACAAGCAAAATAAAATACGTTCGCTC	Confirm integration of *SED*1$_{P}$-SS-
**J-cbh2-check-R**	AATGCATATACGGTTAGCTCATGGTATG	*C.l.cbh2*-AD-*DIT*1$_{T}$ in transformants

Table S3 Yeast strains constructed in this study with their respective collection numbers at the Biobanks South Africa Yeast Culture Collection at the Department of Microbiology and Biochemistry, University of the Free State.

S/N	Accession Form Nr	UFS Collection Nr	Other Nr (Strain Nr)
S. cerevisiae **MH1000 transformed with** ***T.r.EG2, A.a.BGL1, T.e.CBH1*****, &** ***C.l.CBH2***	3070	UOFS Y-3424	MH1000_BECC/MH1000_V1
S. cerevisiae **YI13 transformed with** ***T.r.EG2, A.a.BGL1, T.e.CBH1*****, &** ***C.l.CBH2***	3071	UOFS Y-3425	YI13_BECC/YI13_V1
S. cerevisiae **YI59 transformed with** ***T.r.EG2, A.a.BGL1, T.e.CBH1*****, &** ***C.l.CBH2***	3072	UOFS Y-3426	YI59_BECC/YI59_V1

Figure S1: Schematic illustration depicting the strain construction strategy and the engineered metabolic pathways implemented in this study.

A: **B:**

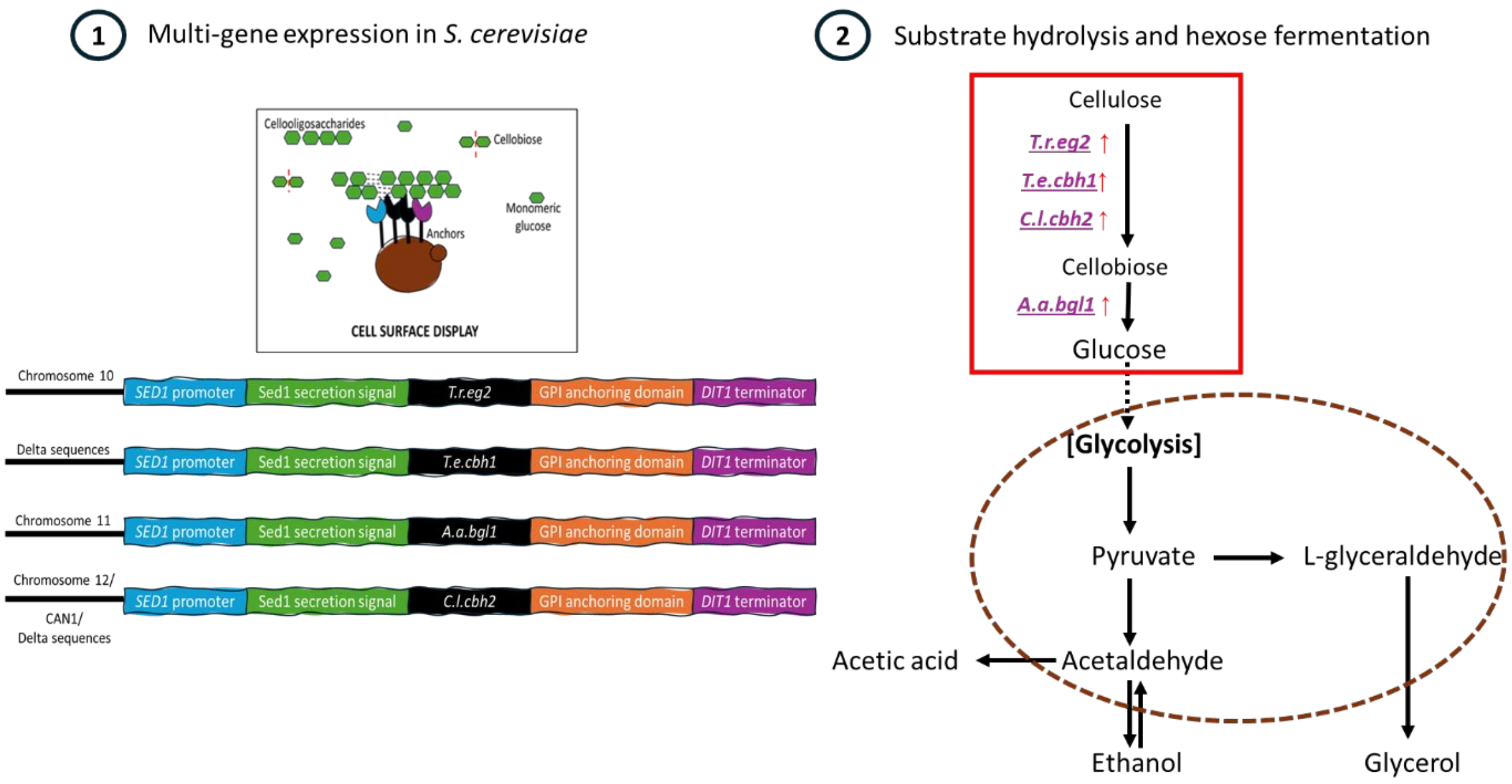

Fig. S1 Schematic illustration depicting the strain construction strategy and the engineered metabolic pathways implemented in this study. (A) the cell surface tethered engineering strategy used, (B) the engineered metabolic module involves heterologous cellulase expression, enabling the hydrolysis of crystalline cellulose into glucose, which can subsequently be assimilated and metabolised by the cell for growth and ethanol production. Please refer to the main text for details of the cellulase genes used.

Figure S2 Enzyme activity for wild-type strains

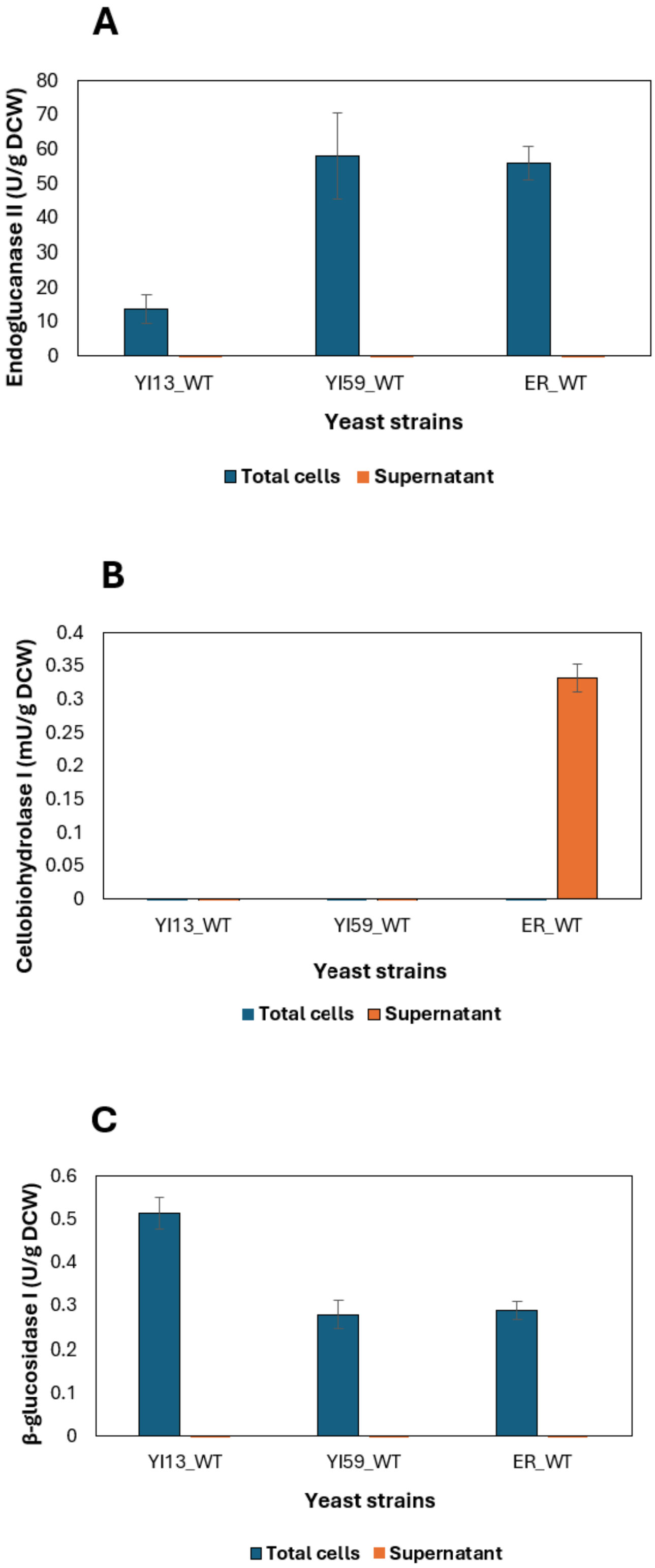

Fig. S2 Background cellulase activities of the parental yeast strains used in this study. (A) endoglucanase activity, (B) cellobiohydrolase activity, (c) β-glucosidase activity. As all of these strains show practically no activity in comparison with transformed strains, this data was omitted from Fig. 1 in the main text.

Figure S2 TEM images

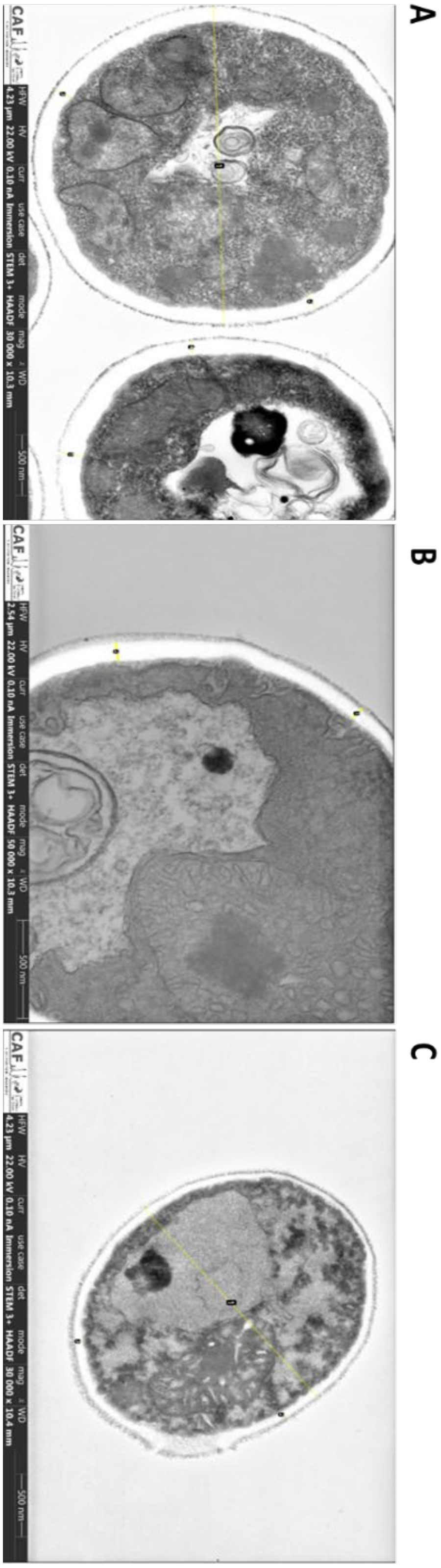

Fig. S3 Cell wall thickness determined with transmission electron microscopy. Yeast strains were cultivated in normal growth conditions until stationary phase. Cells were subjected to microscopic analysis whereby cells were dissected to ultimately determine the thickness of the cell wall. Images show representative individual cells in a population for each of the variant of the YI59 background, namely, (A) YI59_WT, (B) YI59_V1, and (C) YI59_V2

References:

Brevnova E, McBride JE, Wiswall E, Wenger KS, Caiazza N, Hau HH, Argyros A, Agbogbo F, Rice CF, Barrett T, Bardsley JS, Foster AS, Warner AK, Mellon M, Skinner R, Shikhare I, den Haan R, Gandhi CV, Belcher A, Rajgarhia VB, Froehlich AC, Deleault KM, Stonehouse E, Tripathi SA, Gosselin J, Chiu Y, and Xu H. (2011) Yeast expressing saccharolytic enzymes for consolidated bioprocessing using starch and cellulose. Patent No. WO/2011/13516.

Ilmén M, den Haan R, Brevnova E, McBride J, Wiswall E, Froehlich A, Koivula A, Voutilainen SP, Siika-aho M, le Grange DC, Thorngren N, Ahlgren S, Mellon M, Deleault K, Rajgarhia V, van Zyl WH, and Penttila M. (2011) High level secretion of cellobiohydrolases by *Saccharomyces cerevisiae*. *Biotechnology for Biofuels* **4(30).**

Inokuma K, Bamba T, Ishii J, Ito Y, Hasunuma T, and Kondo A. (2016) Enhanced cell-surface display and secretory production of cellulolytic enzymes with *Saccharomyces cerevisiae* sed1 signal peptide. *Biotechnology and Bioengineering* **113(11),** 2358-2366. doi:10.1002/bit.26008

Inokuma K, Kitada Y, Bamba T, Kobayashi Y, Yukawa T, den Haan R, van Zyl WH, Kondo A, and Hasunuma T. (2021) Improving the functionality of surface-engineered yeast cells by altering the cell wall morphology of the host strain. *Applied Microbiology and Biotechnology* **105(14-15)**. doi:10.1007/s00253-021-11440-6

Jacob O, van Lill GR, den Haan R, (2022) CRISPR-based multi-gene Integration strategies to create *Saccharomyces cerevisiae* strains for consolidated bioprocessing. *Applied Sciences* **12(23)**, 12317. doi:10.3390/app122312317
